# The role of AJB35136 and *fdtA* genes in biofilm formation by avian pathogenic *Escherichia coli*

**DOI:** 10.1186/s12917-023-03672-7

**Published:** 2023-08-18

**Authors:** Muhammad Moman Khan, Aamir Ali, Rafał Kolenda, Olugbenga Adekunle Olowe, Jörg Weinreich, Ganwu Li, Peter Schierack

**Affiliations:** 1 Institute of Biotechnology, Brandenburg University of Technology, Cottbus-Senftenberg, Universitätsplatz 1, D-01968 Senftenberg, Germany; 2 National Institute for Biotechnology and Genetic Engineering College, Pakistan Institute of Engineering and Applied Sciences (NIBGE-C, PIEAS) , Jhang Road, POBox 577, Faisalabad, Pakistan; 3 Department of Biochemistry and Molecular Biology, Faculty of Veterinary Medicine, Wrocław University of Environmental and Life Sciences, Wrocław, Poland; 4 Quadram Institute, Norwich Research Park, Norwich, UK; 5 Department of Medical Microbiology and Parasitology, College of Health Sciences, Ladoke Akintola University of Technology, Ogbomosho, Oyo State Nigeria; 6 Department of Veterinary Diagnostic and Production Animal Medicine, College of Veterinary Medicine, Iowa State University, Ames, USA; 7 State Key Laboratory of Veterinary Biotechnology, Harbin Veterinary Research Institute, Chinese Academy of Agricultural Sciences, Harbin, China

**Keywords:** APEC, Biofilm, Gene complementation, Transposon mutant, VideoScan

## Abstract

**Background:**

Infections caused by avian pathogenic *Escherichia coli* (APEC) result in significant economic losses in poultry industry. APEC strains are known to form biofilms in various conditions allowing them to thrive even under harsh and nutrient-deficient conditions on different surfaces, and this ability enables them to evade chemical and biological eradication methods. Despite knowing the whole genome sequences of various APEC isolates, little has been reported regarding their biofilm-associated genes. A random transposon mutant library of the wild-type APEC IMT 5155 comprising 1,300 mutants was analyzed for biofilm formation under nutrient deprived conditions using Videoscan technology coupled with fluorescence microscopy. Seven transposon mutants were found to have reproducibly and significantly altered biofilm formation and their mutated genes were identified by arbitrary PCR and DNA sequencing. The intact genes were acquired from the wild-type strain, cloned in pACYC177 plasmid and transformed into the respective altered biofilm forming transposon mutants, and the biofilm formation was checked in comparison to the wild type and mutant strains under the same conditions.

**Results:**

In this study, we report seven genes i.e., *nhaA*, *fdeC*, *yjhB*, *lysU*, *ecpR*, AJB35136 and *fdtA* of APEC with significant contribution to biofilm formation. Reintroduction of AJB35136 and *fdtA*, reversed the altered phenotype proving that a significant role being played by these two O-antigen related genes in APEC biofilm formation. Presence of these seven genes across nonpathogenic *E. coli* and APEC genomes was also analyzed showing that they are more prevalent in the latter.

**Conclusions:**

The study has elucidated the role of these genes in APEC biofilm formation and compared them to adhesion expanding the knowledge and understanding of the economically significant pathogens.

## Background

Avian pathogenic *Escherichia coli* (APEC), a pathotype belonging to extra-intestinal pathogenic *E. coli* (ExPEC), causes various localized or systemic infections called ‘colibacillosis’ in birds. It is characterized by fibrinous lesions surrounding different organs [1] resulting in colisepticemia, hemorrhagic septicemia, coligranuloma (Hjarre’s disease), air sac disease (chronic respiratory disease), swollen-head syndrome, venereal colibacillosis, coliform cellulitis (inflammatory or infectious process), peritonitis, salpingitis, orchitis, osteomyelitis/synovitis (including turkey osteomyelitis complex), panophthalmitis, omphalitis/yolk sac infection, and enteritis [2, 3]. APEC adversely affects all stages of production and all sectors of the poultry industry including broiler and egg laying hens commencing in high morbidity and mortality leading to significant economic losses [4]. Poultry serves as a host for APEC and consumption of infected undercooked meat can lead to zoonotic transmission in humans becoming potential reservoirs for the pathogen [5]. APEC are responsible for substantial economic losses possibly due to bacterial biofilms and associated resistance to antimicrobials making them highly resilient to the eradication and suppression strategies [1]. APEC share the common virulence factors with UPEC, thus linked to certain diseases in humans [3, 4].

Biofilms are well-defined as a population of bacterial cells that live in a matrix of self-produced exopolysaccharides (EPS), proteins and DNA known as extracellular polysaccharide matrix (EPM), contributing in their attachment and colonization to various inert and living surfaces [6–8]. Biofilm formation is a multi-step process beginning with the initial attachment of microbial community to the surface and then progressing on to detachment [9]. The biofilm EPM contributes not only in the cell to cell adhesions but also acts as a barrier to protect the bacterial community against the host’s defense systems [10]. Therefore, biofilms allow the bacterial cells to endure and persist against harsh environmental, chemical and antimicrobial pressures [11]. A number of acute and chronic infections are related to biofilm-associated drug resistance and tolerance [12–15].

Complete genomic sequencing of APEC strain (APEC O1:K1:H7) has revealed that the functions of a large number of genes are still unknown while the roles of certain genes in various steps of its pathogenesis are still hypothetical and speculative [16]. Biofilm formation in APEC and its role in infections and pathogenesis have not been well studied. Several qualitative and quantitative methods have been developed for the detection of biofilms in laboratory [17]. Qualitative test for the detection of biofilm formation includes Congo Red agar test and pellicle formation assay, whereas the quantitative analysis of biofilm includes microtiter plate assay with crystal violet (CV) staining [18]. As an alternative, the VideoScan technology promises a direct, rapid and automated detection and quantification of fluorescent objects [19], we have successfully used this technology for detection and quantification of biofilms formed by different *E. coli* pathotypes [20], *P. aeruginosa* [21] and *Salmonella* [22]. The current study applied this direct approach and screened and quantified biofilm formation of 1,300 transposon mutants of a previously reported library of a wild-type APEC strain, IMT 5155 [23]. The objective of this study was to identify the APEC genes involved in biofilm formation contributing to the understanding of mechanisms involved in this complex process of colonization and pathogenesis.

## Results

### Screening of transposon mutant library and identification of potential genes involved in biofilm formation

**Fig. 1:**
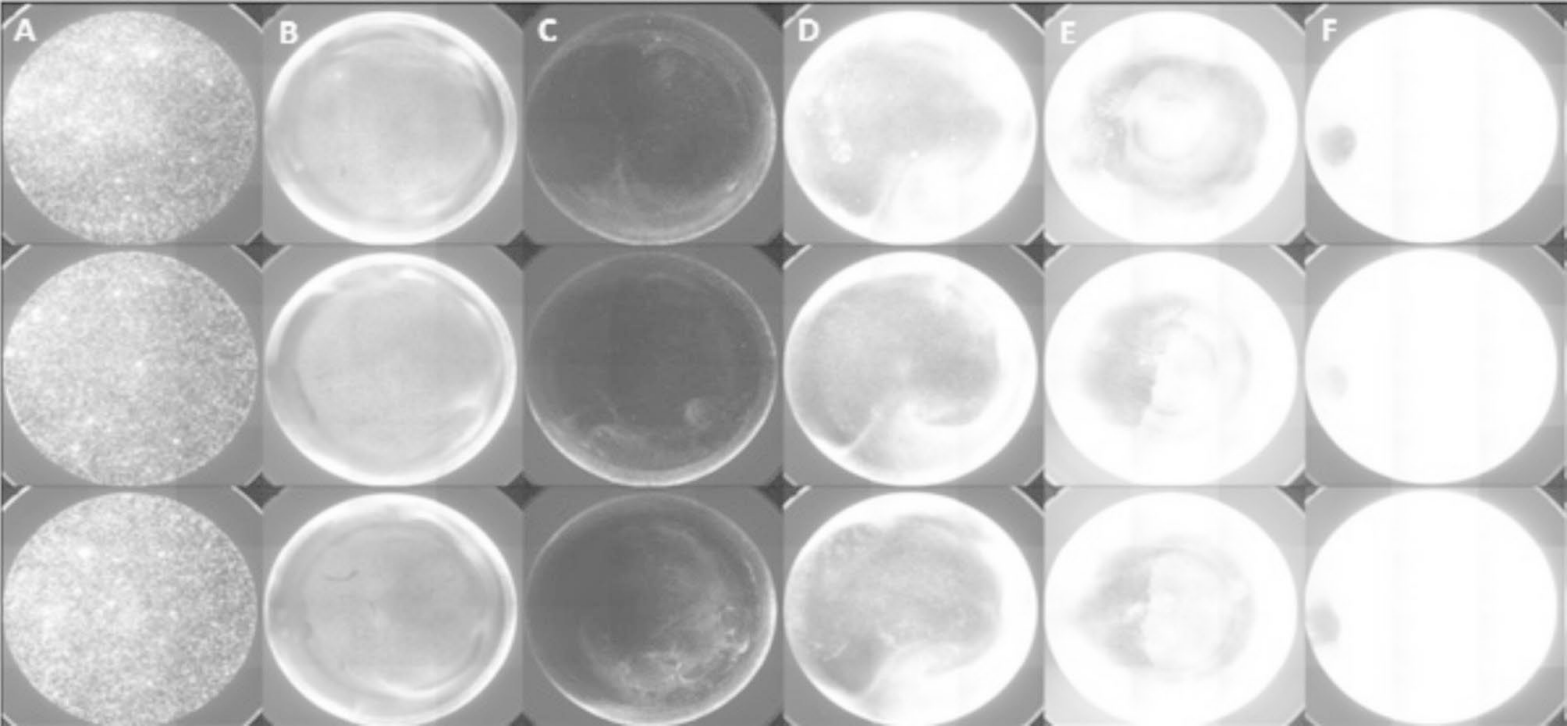
Overview of image of screening of transposon mutants with SYTO 9-stained biofilms. (**A**) plastic beads as internal reference, (**B**) wild-type strain IMT-5155 forming biofilm, (**C**) transposon mutant with down-regulated biofilm formation, (**D**) transposon mutant depicting slightly up-regulated biofilm formation, (**E**) transposon mutant exhibiting up-regulated biofilm formation and (**F**) transposon mutant with highly up-regulated biofilm

A VideoScan module already established and published by Schiebel et al. (2017) was used [20] with minor modifications to quantify the biofilm formation by bacteria (Fig. 1).

By applying this method, 1,300 transposon mutants were screened for biofilm formation in M9 minimal media at 37 °C after 72 h. Transposon mutants (n = 7) with reproducible (six wells with three independent experiments) increase or decrease in biofilm formation under the same conditions as the wild-type strain were branded as “biofilm-altered mutants”. These specific mutants were also subjected to growth assay which revealed that no impairment in growth rate. Sequencing of arbitrary PCR amplicons revealed the identity of the mutated genes in biofilm-altered mutants potentially contributing to biofilm formation (Table 1).

**Table 1:** Transposon mutants with their respective disrupted genes in biofilm-altered mutants

Mutant name	Gene name/ locus	Complete name	(Possible) role/function	PSORT (location)	Biofilm Formation
A10k	*nhaA*	Na^+^/H^+^ antiporter *NhaA*	Na^+^/H^+^ antiporter	Cytoplasmic membrane	Down
A7r	*fdeC*	Intimin-like adhesin *FdeC* (factor adherence *E. coli*)	Adhesin	Outer membrane	Down
C4s	*yjhB*	MFS transporter	Probably sialic acid transport	Cytoplasmic membrane	Up
D11r	*lysU*	lysine—tRNA ligase [multifunctional]	aminoacyl tRNA synthetases	Cytoplasmic	Up
D2q	*ecpR*	Helix-turn-helix transcriptional regulator/putative fimbrial transcriptional regulator	ECP transcriptional regulator	Unknown	Down
E4r	AJB35136	Group 1 glycosyl transferase	Probably O-antigen synthesis	Cytoplasmic	Up
H6n	*fdtA*	TDP-4-oxo-6-deoxy-alpha-D-glucose-3,4-oxoisomerase	O-antigen synthesis	Cytoplasmic	Down

### Motility assays reveal contribution of selected biofilm-altered mutants to APEC motility

**Fig. 2:**
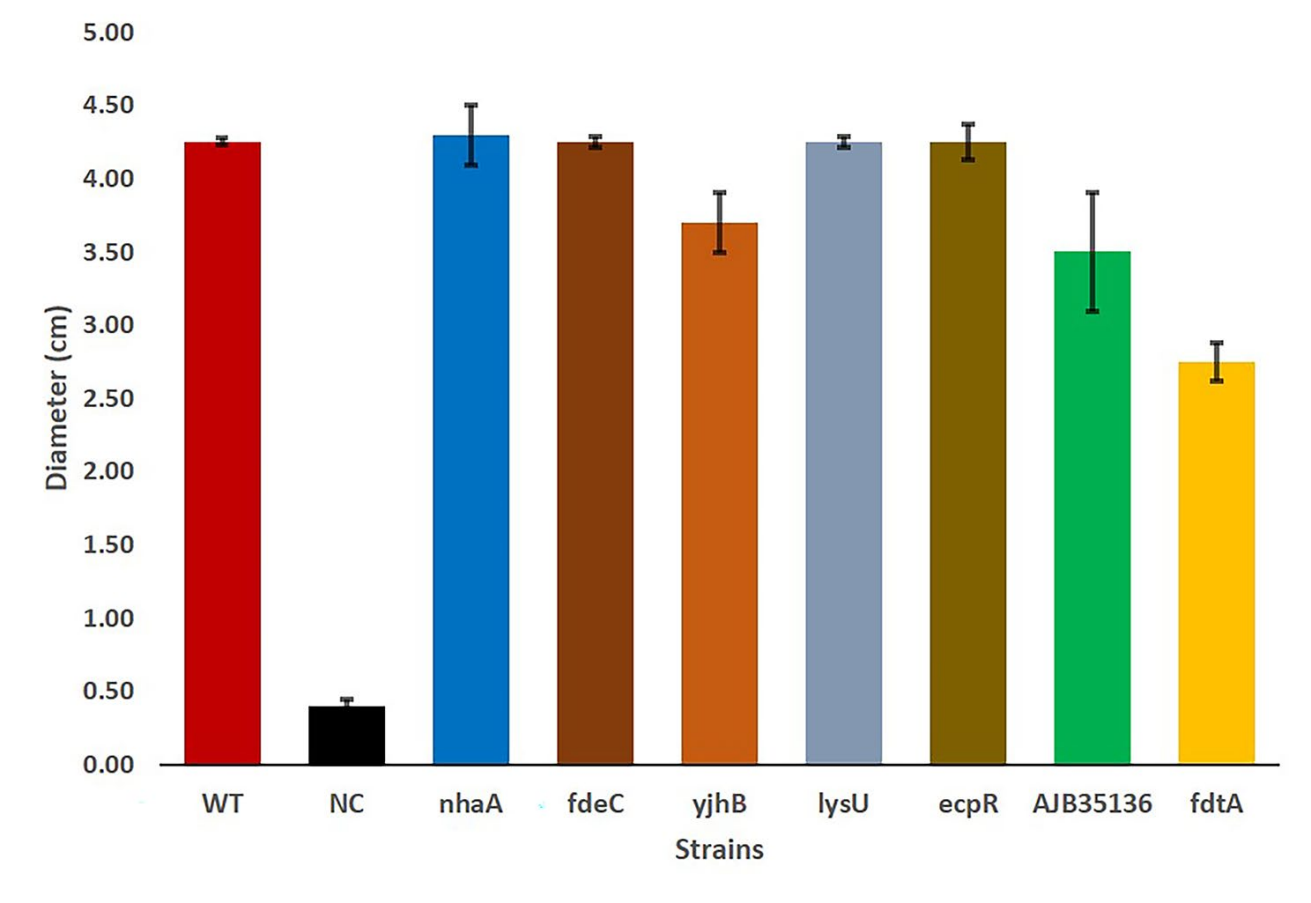
Motility assay of APEC wild-type strain IMT5155 and selected transposon mutants. The data are shown as median values and standard deviation of three separate experiments. SAEC5148, non-motile strain used as a negative control

The results of the motility assay of biofilm-altered mutants in comparison to WT are shown in Fig. 2. One mutation which partially affected O-antigen synthesis (*fdtA*) showed more than 30% reduction in motility compared to the wild type. The *yjhB* (MFS transporter) and group 1 glycosyl transferase (GenBank accession no. AJB35136) mutants showed 4–18% lower motility in comparison to the wild type. Gene mutants such as *nhaA*, *fdeC*, *lysU* and *ecpR* had no significant difference in motility when compared to the wild-type strain.

### Complementation of intact genes from wild-type APEC IMT5155 confirms contribution of biofilm-altered mutants to APEC biofilm formation

**Fig. 3:**
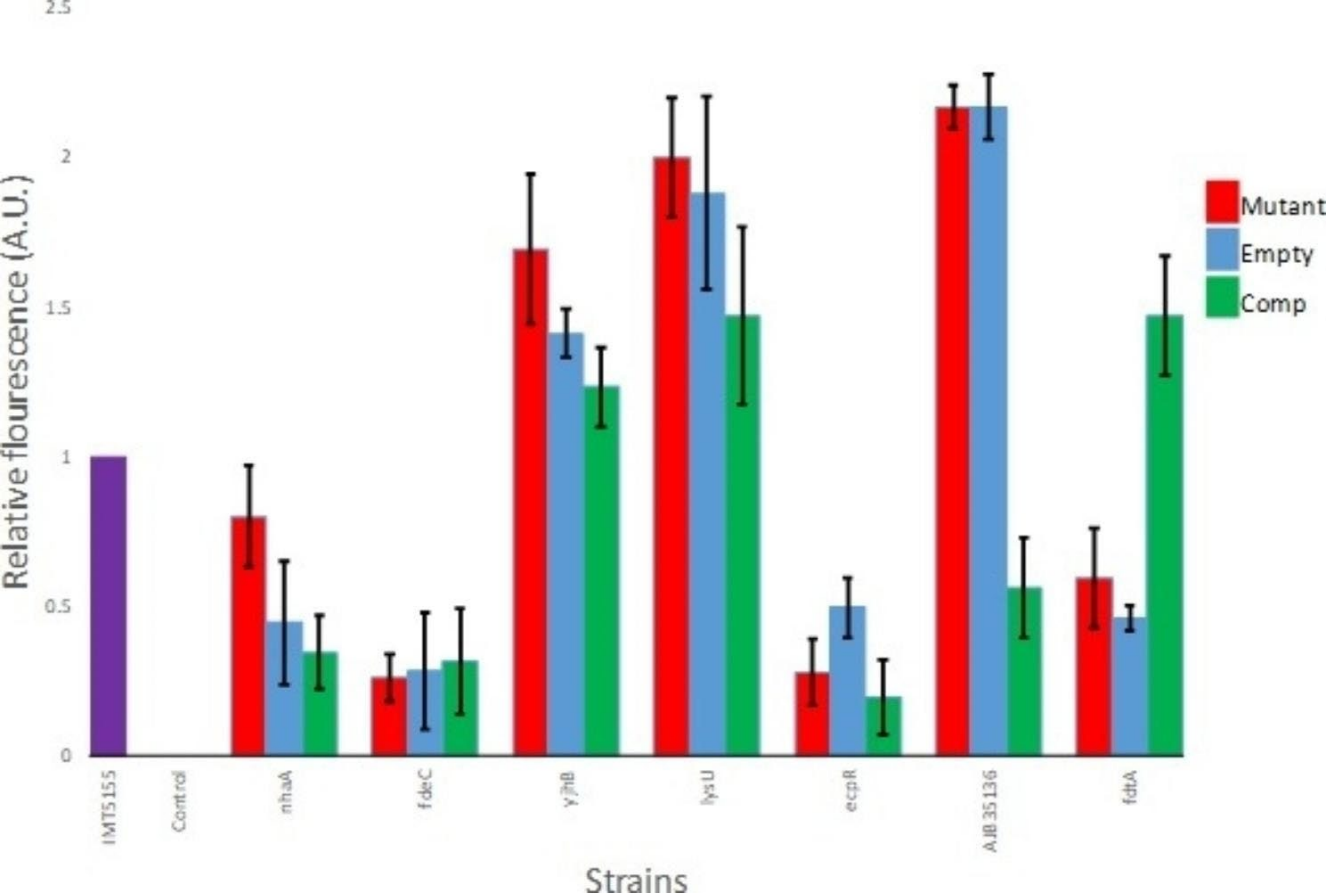
Biofilm formation. APEC wild-type strain IMT5155 (purple bar), selected transposon mutants (Mutant, red bar), and transposon mutants transformed with either empty vector pACYC177 (empty, blue bar) or pACYC177 plasmids bearing wild-type versions of transposon mutated genes (Comp, green bar) in M9 minimal media at 37 °C after 72 h. The data shown are mean values and standard deviation of three separate experiments in six wells replicate for each experiment. Wells not incubated with bacteria were used as a negative control

All biofilm-altered mutants when tested for susceptibility to ampicillin before complementation were found sensitive. The effect of complementation on bacterial growth was also analyzed in “complemented biofilm-altered mutants” via growth assays and no change in growth was detected. Biofilm assay for the screening of biofilm-altered mutants and complemented biofilm-altered mutants exhibited significant complementation effects for two of the genes identified in the original screen i.e., AJB35136 and *fdtA* (Fig. 3). Transformation of biofilm-altered mutants with only the empty vector plasmid alone exhibited no significant change for these mutants in biofilm formation. The complementation of *fdtA* resulted in biofilm formation comparable to that of the wild-type strain whereas its mutant formed really weak biofilm, proving its important role in this complex process. In case of complementation by AJB35136, drastic decrease of around 74% in biofilm formation was observed when compared to its mutant whose biofilms was twice as that of the wild-type strain. For Na^+^/H^+^ antiporter *nhaA* gene and MFS transporter *yjhB* gene, their complementation although decreased biofilm formation but this decrease was not significant as the mutant transformed with empty plasmid also affected biofilm formation. Both *fdeC* and *ecpR*, complementation with intact gene or empty plasmid did not cause any alteration in biofilm forming ability of these strains (Fig. 3).

### Genes from biofilm-altered mutants have higher prevalence in APEC compared to nonpathogenic ***E. coli***

**Fig. 4:**
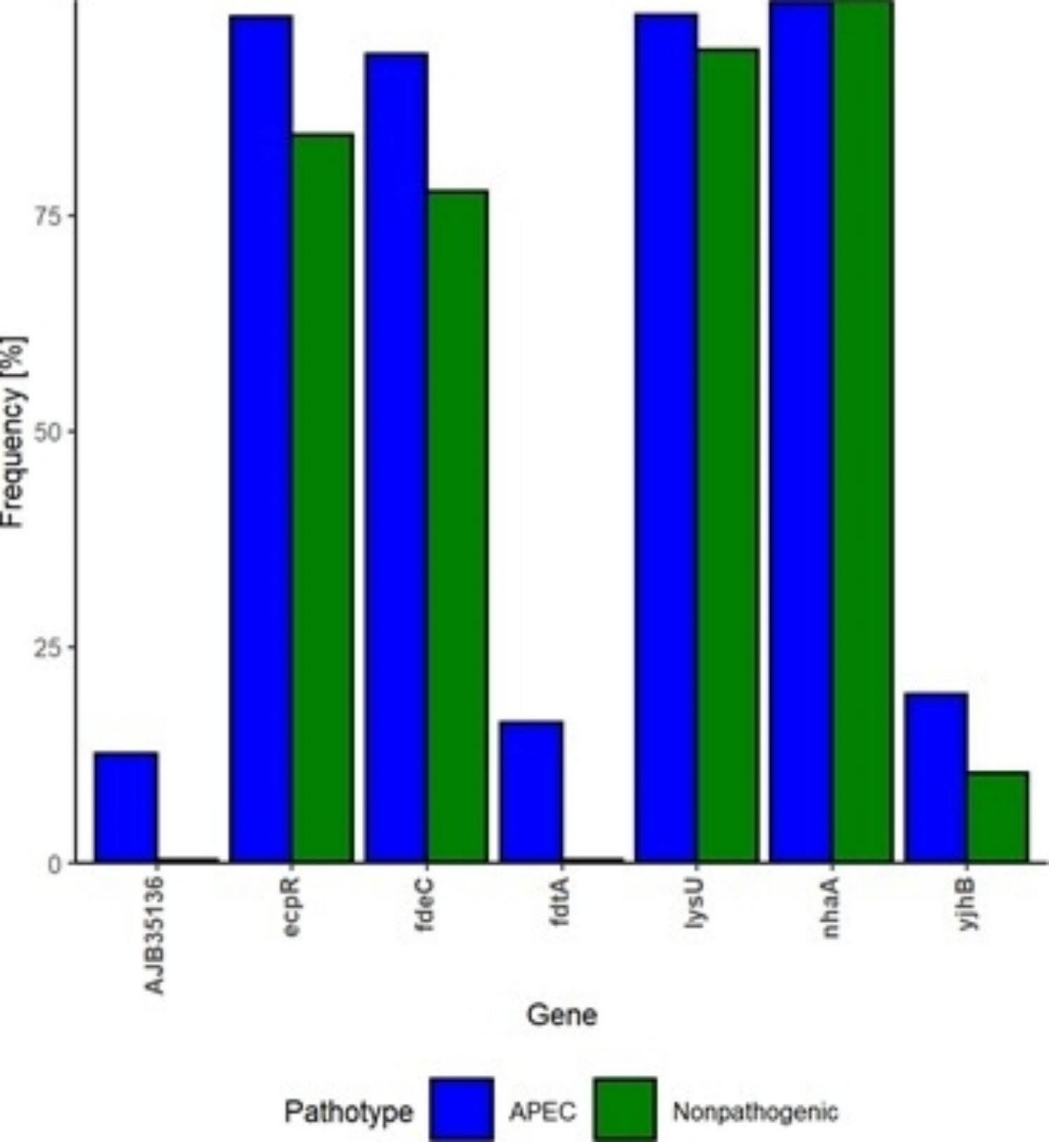
Prevalence of the selected genes in APEC and nonpathogenic *E. coli*. Gene prevalence/frequency percentage (y-axis) is shown for selected genes (x-axis) for the biofilm altered mutants. The analysis was carried out on 573 APEC and 3,069 nonpathogenic *E. coli* genomes

The prevalence of selected genes was carried among two groups to evaluate their frequency in *E. coli* present in nature as depicted in Fig. 4. Genes such as *ecpR, fdeC* and *lysU* were prevalent in higher number in both APEC and nonpathogenic *E. coli* but they were significantly more frequent in APEC genomes (p.BH < 0.0001). The genes which were less frequent in APEC (i.e., *fdtA* (16.23%), AJB35136 (12.57%) and *yjhB* (19.55%) were significantly (p.BH < 0.0001) scarcer in nonpathogenic *E. coli* (i.e., *fdtA* (0.36%), AJB35136 (0.33%) and *yjhB* (10.43%). Only *nhaA* gene was present across both APEC and nonpathogenic *E. coli* with similar prevalence (99%).

## Discussion

Among pathogenic *E. coli*, APEC is a major bacterial pathogen of poultry with considerable zoonotic importance and can potentially serve as a virulence gene reservoir [24]. APEC strains generally form biofilms on different surfaces such as polystyrene, polypropylene, polyvinylchloride (PVC) under scarce nutrient conditions and different temperatures [1, 20] and these conditions can be easily encountered in commercial poultry facilities and farms in water systems, drinkers, feeding trays etc. [1]. APEC biofilm formation has been previously investigated but the focus has been on different biofilm formation types, suitable media [25, 26], characterization of strains from colibacillosis cases [27] along with other factors such as antibiotic resistance and virulence factors [28]. So far, the role of various genes in biofilm formation of APEC remains elusive and therefore a transposon mutant library of 1,300 APEC mutants was screened and selective intact genes were introduced or complemented in the mutants to evaluate their role in this complex process. Here, we used our already established method which is specific, rapid, and high-throughput to detect and quantify the biofilm formation [20].

Most of these genes have also been previously reported in our previous study where the same transposon library was analyzed for adhesion to CHIC-8E11 and Lovo cell lines and similar strategy was used whereby complementation of the mutated genes was carried out [29].

The gene *fdtA* encodes iTDP-4-oxo-6-deoxy-alpha-D-glucose-3,4-oxoisomerase involved in O-antigen synthesis and its corresponding transposon mutant (H6n) exhibited reduced adhesion previously, and reduced biofilm formation (almost half of that of wild type) in the current study. After complementation, a substantial increase in its adhesion property had been reported, now similarly its biofilm formation has also been found significantly increased and became at par with the wild type [29–31]. This showed that *fdtA* is not only involved in adhesion and surface to surface contact with eukaryotic cells but it is also potentially involved in initial attachment and colonization on non-living surfaces. In case of AJB35136 (encoding group 1 glycosyl transferase), it is involved in lipopolysaccharide biosynthesis and the corresponding transposon mutant had low adhesion to the cell lines [29]. It contrasts with our current findings of biofilm formation where the transposon mutant produced more than two-fold high biofilm under the tested conditions as compared to wild-type strain. The reintroduction of this gene decreased biofilm formation significantly and the biofilm became almost half of that of the wild type under same conditions. On biofilm formation, the opposite effect of complementation of both O-antigen synthesis related genes points towards the diversity in nature of genes, their expression and other complex underlying mechanisms determining their roles.

For other genes, complementation did not significantly affect biofilm formation such as *yjhB nhaA*, *lysU, fdeC* and *ecpR* when compared to wild type, although their transposon mutants differed both in adhesion and biofilm formation phenotypes. The transposon mutant of *yjhB* gene produced high biofilm and similarly the adhesion percentage on Lovo and CHIC-8E11 cell lines was also very high when compared to the wild-type strain [29]. In both cases, reintroduction of the same gene in the mutant did not have any significant effect on either of the phenotype. *yjhB* has been identified as putative sialic acid transporter and has been suggested to indirectly effect the regulation of expression of type 1 fimbriae [32, 33]. It can be concluded that it plays a role in both virulence attributes of adhesion and biofilm formation but the role has to be further confirmed by complementation via an appropriate expression system. A previous study showed that a mutation in *nhaA* gene encoding the Na/H antiporter in *E. coli* K-12 strain had increased motility and FliC protein expression [34], this may have translated into high adhesion [29] but did not significantly boost biofilm formation. Also the *nhaA* transposon mutant did not exhibit significantly high motility and this is in contrast to the previously reported study [34]. For motility assays, Smith et al., (2017) used YESCA agar and incubation temperature of 26 ºC, whereas LB agar and 37 ºC incubation were used in the current study. As it has been shown previously that media or temperature differences can effect gene expression of *E. coli*, the contradiction regarding the effect of *nhaA* mutation on motility could be due to methodological differences in design of motility experiments [35, 36].

Another interesting gene not identified in the previous study with its transposon mutant exhibiting really high biofilm formation under the above-mentioned conditions was *lysU* gene. The reintroduction of intact *lysU* gene in the transposon mutant D11r did not result in significant decrease in the biofilm formation. *lysU* has been identified as heat inducible lysyl-tRNA synthetase of *E. coli* and its deletion has been shown to grow adequately under normal conditions but the growth is hampered at higher temperatures (i.e., 44 °C) [37]. The partial effect of complementation of *lysU in trans* might be associated with the pACYC177 plasmid system used in this study. This system has been used successfully in multiple species in the past [36, 38]. The complemented mutant exhibited tendency to lower biofilm formation, but relatively high level of variation was observed indicating that other complementation systems e.g. inducible expression plasmids pBAD33 or pWRG30 could provide better results in further experiments for this gene.

The *fdeC* (encoding intimin-like adhesion) transposon mutant exhibited really low biofilm formation in contrast to the slightly high adhesion to the cell lines and complementation through pACYC177 did not result in any significant change in biofilm formation or adhesion on either of the cell lines. Contribution of FdeC to biofilm formation of STEC has been shown in study of Easton et al. [39] but the expression conditions were the crucial factor for FdeC contribution. Our gene frequency analysis revealed lower abundance of *fdeC* gene in nonpathogenic *E. coli* than in APEC. This result is in agreement with a previously reported study, where it showed the conservation of FdeC among strains of different *E. coli* pathotypes and elicit protection against urinary tract infections [40]. The *ecpR* transposon mutant i.e., D2q had very high adhesion rate and upon complementation decreased 3 folds and became similar to wild type only in case of CHIC-8E11 cell line. In case of biofilm formation, this mutant produced very low biofilm and complementation did not have any significant effect when the same system of complementation was used. The *ecpR* gene encodes the *E. coli* common pilus (ECP) regulator is a part of the ECP gene cluster [41].

In summary, we report the identification of 7 genes affecting biofilm formation of APEC under stressed conditions with low nutrition. This study also further analyzes the role of some of the previously identified genes effecting adhesion phenotype, evaluates their effect on biofilm formation and compares their influence on both phenotypes. Both are important in colonization and pathogenesis of infectious bacteria as adhesion deals with initial contact and colonization of bacteria to the living tissue and host cells and biofilm formation was studied as in this case it also involves initial attachment and colonization on a non-living surface. The study also shows that same transposon mutants show different trend in the two virulence mechanism depicting diverse role of these genes under different situations.

It is also important to note that when intact genes were cloned in pACYC177 plasmid and reintroduced in intact form into the transposon mutant, it did not result in complementation for all the cases. Therefore, the effect on phenotype holds true for only two genes i.e., *fdtA* and AJB35136 where the empty plasmid did not alter the biofilm formation and only the plasmid cloned with intact gene showed increase or decrease in the biofilm formation. For all the other genes, complementation did not significantly affect the phenotype under consideration. Similar issues were observed in study of Young et al., (2022), where complementation of APEC O18 deletion mutant using pBAD33 did not alter the biofilm formation [42]. This can be due to the nature of genes which needs to be expressed at a specific time point or under an inducible promoter instead of the constitutive promoter and points to a complex underlying mechanisms of biofilm formation which requires further research. This study contributes toward the understanding of APEC biofilm formation and compares the roles played by the same genes in adhesion.

## Conclusion

APEC adversely affects all stages and sectors of poultry industry commencing in high morbidity and mortality leading to significant economic losses. This study identified seven genes that play a significant role in the biofilm formation of APEC under nutrient deprived conditions. Two O-antigen related genes, AJB35136 and *fdtA*, have a particularly important contribution to biofilm formation and regulation in APEC. This research provides valuable insights into the mechanisms of biofilm formation in APEC and may contribute to the development of effective strategies to control and prevent APEC persistence in the poultry industry. As phages provide useful alternative as surface decontaminants, targeting genes that also reduce biofilm formation with phages in the future might provide valuable resource in reduction of APEC burden in poultry production.

## Materials and methods

### Bacterial Strains

Wild-type clinical isolate APEC IMT 5155 along with its transposon mutant library of 1,300 random transposon mutants (coding Kanamycin resistance cassette) were acquired from Free University (FU) Berlin, Germany [23]. The mutants were revived on LB (Lysogeny Broth) agar plates with the appropriate antibiotic i.e., kanamycin (50 μg/ml) whereas the APEC IMT 5155 was revived on an antibiotic free CHROM agar plate (Mast Diagnostica GmbH, Reinfeld, Germany).

### Screening of transposon mutant library for biofilm formation

Initially, wild-type APEC IMT 5155 was subjected to various biofilm forming conditions including four growth media i.e., brain heart infusion broth (BHI), LB broth, tryptic soya broth (TSB) and M9 minimal media; two incubation temperatures i.e., 28 and 37 °C, and three variable durations: 24, 48 and 72 h. The biofilms were quantified with our previously optimized protocol [20] with minor modifications. Since the wild-type APEC IMT 5155 formed adequate biofilm in M9 minimal media at 37 °C after 72 h, therefore the 1,300 transposon mutants were screened under these conditions and the up/down regulation of biofilm formation was evaluated in 96-well flat bottom polypropylene plates (Greiner Bio-One GmbH, Frickenhausen, Germany) to make biofilm [20]. *E. coli* strain K-12 MG1655 F’tet Δ*traD* was used as biofilm forming positive control. The plates were covered with sealing films and incubated at 37 °C for 72 h. The non-adherent bacteria from the wells were aspirated and attached biofilms were washed once with 200 μl of sterile 0.9% NaCl followed by an incubation in the dark for 10 min with isotonic saline containing 5 μM SYTO 9 green fluorescent nucleic acid stain (Thermo Fisher Scientific GmbH, Dreieich, Germany). The plates were washed again with isotonic saline and analyzed using the automated VideoScan technology platform. Biofilm formation potential of each mutant was checked in triplicate wells with two independent experiments. The mutants suspected for up/down regulation of biofilm formation were further tested in six wells with three independent experiments and the mutants showing significantly up/down regulation in biofilm formation as compared to wild-type strain were designated as ‘biofilm-altered mutants’. The shake flask growth studies of the wild-type strain and biofilm-altered mutants were done at 37 °C to rule out any effect of transposon mutation on the bacterial growth [29].

### Identification of transposon insertion sites in ‘biofilm-altered mutants’

The ‘biofilm-altered mutants’ were subjected to arbitrary polymerase chain reaction (PCR) using Ampli *Taq* Gold polymerase to locate the transposon cassette insertion site and identify the mutated genes [29]. The products of first round PCR were amplified again in another round/nested PCR using one arbitrary primer (Arbi2, as homologous to the 5^/^ sequence of Arbi5) and one transposon I terminus-specific primer (P6) as described earlier [23]. Sequences of all the primers used in the study are shown in Table 2. Genomic DNA of the wild-type APEC, and a reaction without template were used as templates for negative controls. The amplicons were purified with PCR purification kit (Thermo Scientific) and sent for commercial Sanger sequencing (LGC’s Agova Genomics, Berlin, Germany). The DNA sequences were analyzed in NCBI public databases using BLASTn and bacterial localization prediction tool (PSORTb 3.0) [43] and the genes mutated by transposon insertions and potentially involved in regulation of biofilm formation were selected for further experiments.

**Table 2:** List of primers used in the study

Gene name/Target Sequence	Primer Name	Sequence 5’to 3’	
**Primers for arbitrary PCR**			
Flanking sequence Tn5	P9	CGCAGGGCTTTATTGATT	
Arbitrary	Arbi5	GGCCACGCGTCGACTAGTAC(N)10TACNG	
Flanking sequence Tn5	P6	CCTAGGCGGCCAGATCTGAT	
Arbitrary	Arbi2	GGCCACGCGTCGACTAGTAC	
**Primers for amplification of genes from wild-type APEC**			
nhaA	A10kFwd	ACATAAGCTTTTGACAATTAATCATCGGCTCG TATAATGTGTGGAGGAGGACAGCTATGAAAC ATCTGCATCGATTCTTTAGC	
	A10kRev	ACATAAGCTTTCAAACTGATGGACGCAAACGA ACGCGTAACCAGC	
fdeC	A7rFwd	ACATGGATCCTTGACAATTAATCATCGGCTCG TATAATGTGTGGAGGAGGACAGCTATGTCACA TTATAAAACAGGTC	
	A7rRev	ACATGGATCCCTATTGCTGGGTAAGAGGC	
*yjhB*	C4sFwd	ACATGGATCCTTGACAATTAATCATCGGCTCG TATAATGTGTGGAGGAGGACAGCTATGGCAAC AGCATGGTATAAAC	
	C4sRev	ACATGGATCCTCATTTAGCCACGGATAG	
lysU	D11rFwd	ACATGGATCCTTGACAATTAATCATCGGCTCGTATAATGTGTGGAGGAGGACAGCTATGTCTGAACAAGAAACACGGGG	
	D11rRev	ACATGGATCCTTATTTCTGTGGGCGCATCG	
ecpR	D2qFwd	ACATGGATCCTTGACAATTAATCATCGGCTCGTATAATGTGTGGAGGAGGACAGCTATGGAATG TCAAAACCGTTCTG	
	D2qRev	ACATGGATCCTTACTGAACCAACTTATATATTTTTGAGTACAGC	
AJB35136	E4rFwd	ACATGGATCCTTGACAATTAATCATCGGCTCGTATAATGTGTGGAGGAGGACAGCTATGGAAG AAAATAATATGAAGACG	
	E4rRev	ACATGGATCCTTAATAAATAGATTCATACATA GC	
fdtA	H6nFwd	ACATGGATCCTTGACAATTAATCATCGGCTCG TATAATGTGTGGAGGAGGACAGCTATGGATAT TAAATTAATCTCTTTGC	
	H6nRev	ACATGGATCCTTATGAATTCTCAATTGAATTTA CTCTTC	
Insert sequencing after cloning	pACYC177Fwd	GTAGCGGTTCGGTTTATTGAC	
	pACYC177Rev	TGTCCACGGTACGCCTGC	

### Cloning of intact genes from wild-type APEC IMT 5155 into the pACYC177

The genes selected by arbitrary PCR genes were amplified in intact form from the wild-type APEC IMT 5155 strain by PCR using *Phusion* polymerase (Thermo Scientific, Germany) with primers designed for cloning in expression vector pACYC177. The PCR products were purified by PCR purification kit (Thermo Scientific, Germany) while the pACYC177 plasmid was isolated using the Midiprep Kit (Qiagen N.V., Venlo, NL) and DNA concentrations were measured with a Colibri Microvolume Spectrometer (Titertek-Berthold). The purified PCR products (1 μg) and plasmid DNA (4 μg) were digested with Fast Digest (FD) *BamHI* enzyme (Thermo Scientific) and purified by PCR purification kit (Thermo Scientific) and gel extraction kit respectively (Qiagen N.V., Venlo, NL). The digested and purified PCR products and plasmid were ligated by T4 DNA ligase (Thermo Scientific) following the manufacturer’s protocol for 30 min and transformed by heat shock at 42 °C for 45 s into already prepared *E. coli* XL1Blue chemocompetent cells [44]. The transformants were plated onto LB agar with Kan (50 μg/ml) for selection and incubated at 37 °C overnight and the colonies were tested with the rapid colony screening protocol [45]. The positive colonies were processed for overnight culture in 10 ml of LB broth with Kan (50 μg/mL) and plasmid was isolated by Miniprep plasmid extraction kit (Thermo Scientific) after 16–18 h growth and quantified. To confirm successful cloning, plasmids were purified and digested with *BamHI* and the plasmids, that showed two bands of desired sizes after restriction, were sent for Sanger sequencing commercially (LGC’s Agova Genomics, Berlin, Germany). The plasmid sequences were analysed and clones with accurate gene with promotor sequence and open reading frames were stored at -20 °C.

### Complementation of the ‘biofilm-altered mutants’ with intact genes

The transposon mutants with altered biofilm forming abilities were complemented with intact genes taken from wild-type strain. For complementation, electrocompetent cells of each of the ‘biofilm-altered mutant’ were prepared according to the established protocol [46] with minor modifications. Briefly, LB medium (100 ml) was inoculated with 1 ml of overnight culture and incubated at 37 °C at 180 rpm until OD_600_ = 0.6 was achieved. The bacteria were then incubated on ice for 5 min, centrifuged at 6,000 x *g* for 10 min at 4 °C. The supernatant was discarded and the pellet was washed once with 50 ml of ice cold water and twice with 25 ml of ice cold 10% glycerol in water. Finally, washed bacterial cells were resuspended in 0.4 ml of 10% glycerol in water and aliquoted to 50 μl per tube. All the steps were carried out on ice. For transformation, plasmid (100 ng) was added to particular mutant’s electrocompetent cells, incubated on ice for 1 min, transferred to cuvette (4 mm wide) and electroporated at a voltage of 2.5 kV, with the capacity of 25 μF and resistance of 200 Ω. The bacteria were also transformed with empty plasmid (i.e., without insert) to observe effects of transformation by plasmid only. A volume of 900 μl of super optimal broth media (SOC) was added immediately after transformation and the bacteria were transferred to 2 ml tubes for incubation at 37 °C for 1 h with 180 rpm shaking. The bacteria were then centrifuged at 6,000 x g for 3 min, resuspended in 200 μl of medium, spread onto LB agar plates with ampicillin (100 μg/ml) and kept at 37 °C overnight. The bacterial colonies on agar plates were designated as ‘biofilm-altered mutants complemented with gene of interest’ and ‘biofilm-altered mutants complemented with empty plasmid only’.

To investigate the effect of gene complementation, the wild-type APEC IMT5155 and ‘biofilm-altered mutants’ with and without complementation were checked for their biofilm formation by automated VideoScan technology as described earlier in 6 well replicates and in three independent experiments. The average biofilm formation of wild-type bacteria in each of the 6 wells in three experiments was normalized to 1 and compared for statistical significance with similar treated values of bacteria of ‘biofilm-altered mutants’ with and without complementation with the genes.

### Prevalence of potential biofilm regulating/affecting genes

Genes potentially involved in biofilm formation in the biofilm-altered mutants as selected by arbitrary PCR were checked for their prevalence in the reported APECs and nonpathogenic *E. coli* genomes. A collection of 573 APEC and 3,069 nonpathogenic *E. coli* genomes was obtained from RefSeq as reported earlier [47]. Briefly, when categorizing genomes as nonpathogenic *E. coli*, RefSeq collection of *E. coli* was blasted against virulence associated genes (VAGs) and pathotyped based on presence or absence of VAGs. Genomes encoding none of the VAGs or only one of virulence gene i.e., *fimH*, *fyuA*, *iucC*, *neuC*, *sitA* or *yfcV* were assigned as nonpathogenic *E. coli*. Moreover, genomes lacking information about coverage (except complete genome sequences), coverage less than 50 times, number of contigs more than 400 and 3rd generation sequencing platforms/technologies, i.e., “Oxford Nanopore” and “Pacific Biosciences”, were omitted [47]. APEC genomes were downloaded as assemblies or raw reads from BioProjects found in GenBank and reference APEC genomes. Genome assemblies were made with Shovill pipeline. The prevalence of genes listed in Table 1 in APEC and nonpathogenic *E. coli*, was tested with ABRicate. All results with values of 80% or above were considered positive. The prevalence of genes was compared with chi-squared test of independence [48].

### Motility assays

Initial analysis of the genes mutated in transposon library and their associated literature pointed to their linkage with motility which is known to affect biofilm formation. Therefore, we verified selected strains with transposon mutations with altered biofilm formation and wild-type APEC IMT5155 for motility. The strain SAEC5148 was used as a negative control [29]. The bacteria were inoculated in 1 ml LB medium and incubated overnight at 37 °C with 180 rpm shaking. Each isolate was inoculated into the center of a motility agar plate (LB containing 0.25% agar), and the plates were sealed with paraffin film and kept at 37 °C for 16 h. The diameter of the diffusion zone from the point of inoculation was measured. The diameter of 5 mm or more was considered as motile, whereas less than 5 mm diameter was considered non-motile [20]. The experiments for each of the isolate were carried out three independent times.

## Data Availability

All essential data generated or analyzed during this study are included in this published article.
